# From Agro-Waste to Therapeutic Potential: Spasmolytic Mechanisms of *Vaccinium myrtillus* L. Leaf Extract on Isolated Rat Ileum

**DOI:** 10.3390/plants15030504

**Published:** 2026-02-06

**Authors:** Nemanja Kitić, Katarina Šavikin, Dušanka Kitić, Miloš Jovanović, Milica Randjelović, Jelena Živković, Bojana Miladinović, Nada Ćujić Nikolić, Nenad Stojiljković, Suzana Branković

**Affiliations:** 1 Department of Physiology, Faculty of Medicine, University of Niš, Ave. Dr. Zorana Đinđića 81, 18000 Niš, Serbiabrankovic.suzana@yahoo.com (S.B.); 2 Institute for Medicinal Plants Research “Dr. Josif Pančić”, Tadeuša Košćuška 1, 11000 Belgrade, Serbia; ksavikin@mocbilja.rs (K.Š.);; 3 Department of Pharmacy, Faculty of Medicine, University of Niš, Ave. Dr. Zorana Đinđića 81, 18000 Niš, Serbia; milos.jovanovic@medfak.ni.ac.rs (M.J.); milica.randjelovic@medfak.ni.ac.rs (M.R.); bojana.miladinovic@medfak.ni.ac.rs (B.M.)

**Keywords:** bilberry leaves, gastrointestinal motility, spasmolytic effect, rat ileum, phenolic acids, flavonoids

## Abstract

Bilberry (*Vaccinium* *myrtillus* L., Ericaceae) is chiefly valued as an edible plant for its berries, widely consumed as a functional food, whereas the leaves, as agro-waste, remain an underutilized natural source of bioactives. The traditional use of *V. myrtillus* leaves is well documented, particularly for managing diabetes and gastrointestinal disorders. However, their potential spasmolytic activity, which could support such uses, remains unexplored. The main objective of this study was to evaluate the spasmolytic potential of *V. myrtillus* leaf extract on the gastrointestinal tract and to elucidate its underlying mechanism of action. The spray-dried 50% hydroethanolic extract of *V. myrtillus* leaves, obtained by double percolation, was analyzed using HPLC-DAD. The analysis revealed phenolic acids, with chlorogenic acid as the major compound, and flavonoids, predominantly isoquercitrin. Spasmolytic activity was tested on isolated rat ileum, and the mechanism of action was monitored using models of spontaneous contractions and acetylcholine-, histamine-, CaCl_2_^−^, Bay K8644-, L-NAME-, ODQ-, apamin-, BaCl_2_^−^, charybdotoxin-, glibenclamide-, TRAM-34-, and quinine-modified contractions. The extract’s activity on isolated ileum strips is primarily mediated via Ca^2+^ channels, cGMP, histamine, and NO pathways. Overall, this study affirms *V. myrtillus* leaves as a valuable source of phenolic compounds with potential for treating spasmodic gastrointestinal disorders.

## 1. Introduction

The genus *Vaccinium* L. (Ericaceae) includes around 450 plant species, comprising terrestrial and epiphytic shrubs and sub-shrubs. These species are distributed across Europe, Central and North America, Central and Southeast Africa, and Asia [1]. Bilberry (*Vaccinium myrtillus* L.), also referred to as European blueberry, huckleberry, or whortleberry, is a wild shrub native to mountainous regions and forests across Europe, Asia, and North America [2,3]. The fruits of several *Vaccinium* species, including *V. myrtillus*, are recognized as rich sources of bioactive compounds, valued in nutrition and pharmacy due to their high content of polyphenols and carotenoids. The predominant bioactive compounds in these fruits are anthocyanins [1]. The anthocyanins of *V. myrtillus* fruit, characterized by delphinidin, cyanidin, peonidin, petunidin, and malvidin as aglycones, predominantly include delphinidin-3-*O*-galactoside and delphinidin-3-*O*-glucoside [2]. According to the European Medicines Agency, preparations of *V. myrtillus* fruits (fructus siccus and fructus recens) have a well-documented long-standing traditional medicinal use for the symptomatic treatment of mild diarrhea, minor inflammations of the oral mucosa, discomfort and heaviness in the legs associated with minor venous circulatory disturbances, and cutaneous capillary fragility [4].

While the health benefits of *V. myrtillus* fruits have been extensively investigated, its leaves, traditionally used in many countries for treating various diseases, represent a valuable medicinal raw material that warrants further investigation [1,3]. Furthermore, *V. myrtillus* leaves are easily available as the agro-waste produced by the berry industry. Their utilization could contribute to more efficient biowaste management and biodiversity preservation, which aligns with the principles of sustainable development [5,6,7]. The medicinal properties of *V. myrtillus* leaves are associated with polyphenol compounds, primarily hydroxybenzoic acid derivatives (gallic, syringic, protocatechuic, and vanillic acids) and hydroxycinnamic acid derivatives (chlorogenic, p-coumaric, trans-ferulic, and feruloylquinic acids). Flavonoids, including the subclasses flavonols, flavanols, and flavonolignans, as well as trace amounts of anthocyanins, are also present. Among individual flavonoids, quercetin heterosides are the most common. Beyond polyphenols, minor amounts of phytosterols and triterpenes are found [1,3,8]. Interestingly, it has been demonstrated that *V. myrtillus* leaves contain significantly higher levels of polyphenol compounds compared to the fruit, including total polyphenols, phenolic acids, flavonols, polymeric proanthocyanidins, mono-, di-, and oligomeric flavan-3-ols, and dihydrochalcones, with the exception of anthocyanins [2,9].

In traditional medicine, herbal preparations derived from *Vaccinium* species are most commonly used to treat disorders related to the digestive, endocrine, and genitourinary systems, owing to their astringent, antiseptic, diuretic, and tonic properties [7,8,10]. Leaves of *V. myrtillus* were one of the most frequently used herbal antidiabetic preparations prior to the discovery of insulin. However, results from numerous animal and clinical studies are contradictory and do not fully support this traditional use [7,11]. Empirically, aqueous extracts of *V. myrtillus* leaves are considered particularly effective against bacterial infections and inflammation of the oral cavity [7]. There are reports that *V. myrtillus* leaves have been traditionally used in France as a spasmolytic (i.e., antispasmodic) agent, but such pharmacological effect has not been validated to date [10]. Numerous preclinical studies confirm the multiple therapeutic effects of *V. myrtillus* leaves, including antioxidant and antimicrobial activity, metabolic modulation, and dermatological effects [3].

The global prevalence of gastrointestinal disorders is approximately 40%, with conventional pharmacotherapy being both prolonged and economically demanding [12]. Antispasmodic drugs are commonly used for the symptomatic treatment of gastrointestinal disorders accompanied by smooth muscle cramping.

Recent research supports the use of bioactive compounds from certain medicinal plants, which can exhibit significant spasmolytic effects. These compounds are often more effective, safer, and more cost-efficient compared to synthetic spasmolytic drugs [13]. Although certain bioactive compounds of *V. myrtillus* leaves, such as flavonoids and phenolic acids, may be responsible for the spasmolytic activity of medicinal plants [14], to the best of our knowledge, the spasmolytic activity of preparations from any *Vaccinium* species has not yet been investigated.

Given the lack of pharmacological validation of the traditional use of *V. myrtillus* leaves as spasmolytic agents, this study aimed to evaluate the spasmolytic potential of their extract. Spasmolytic activity was assessed on isolated rat ileum using models of spontaneous contractions and contractions induced by acetylcholine, histamine, CaCl_2_, L-NAME (N*ω*-Nitro-L-arginine methyl ester), ODQ (1H-[1,2,4]Oxadiazolo [4,3-a]quinoxalin-1-one), apamin, BaCl_2_, charybdotoxin, glibenclamide, TRAM-34 (1-[(2-Chlorophenyl)diphenylmethyl]-1H-pyrazole) and quinine to investigate the underlying mechanism of action.

## 2. Results

### 2.1. Phytochemical Profile of the V. myrtillus Leaf Extract (VMLE)

In the present study, the predominant individual polyphenol compounds in the investigated extract were analyzed by HPLC–DAD, and total polyphenol and tannin contents were determined spectrophotometrically, with the results summarized in Table 1. The extract was found to contain significant amounts of caffeic acid derivatives and flavonoid compounds. Among these, chlorogenic acid emerged as the most abundant polyphenol compound, with a concentration of 52.06 ± 1.08 mg/g extract dry weight (dw). The second most abundant compounds were flavonols, exclusively composed of quercetin derivatives, with isoquercitrin prevailing at 35.28 ± 0.83 mg/g dw.

### 2.2. Effects of V. myrtillus Extract on Spontaneous Ileum Contractions

A significant dose-dependent relaxation of the ileum smooth muscle’s spontaneous contractions was observed with the tested extract of *V. myrtillus* (0.005–5 mg/mL). These contractions, although significant, were weaker than those of the nonspecific myorelaxant, papaverine, which was used as a control.

**Figure 1:**
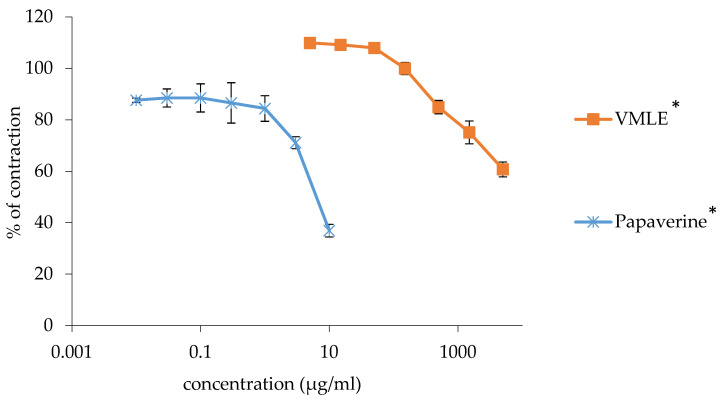
Effects of the *V. myrtillus* leaf extract (VMLE) and papaverine on spontaneous contractions of isolated rat ileum. Contractile responses were normalized to baseline activity recorded in Tyrode’s solution (control, set as 100%) and are presented as mean ± SD from independent animals. Overall effects across the concentration range were evaluated using area under the concentration–response curve (AUC) analysis, with statistical significance assessed using a one-sample Student’s *t*-test (* *p* < 0.05).

Papaverine, at a maximum applied concentration of 0.01 mg/mL, inhibited 36.89 ± 2.75% of contractions out of 100%, whereas the *V. myrtillus* extract, at a maximum concentration of 5 mg/mL, reduced the contractions by 60.76 ± 2.88%. Figure 1 depicts the dose-dependence of spasmolytic activity of the tested extract and papaverine.

### 2.3. Effects of the V. myrtillus Extract on Potassium Chloride-Induced Ileum Contractions

Contractions induced by the usage of a KCl solution (80 mM) were dose-dependently inhibited by the *V. myrtillus* extract. The extract, at the highest applied dose of 5 mg/mL, reduced the smooth ileum contractions to 70.92 ± 2.83%. Verapamil, with a maximum concentration of 0.005%, was used as a control and also reduced the concentration dose-dependently to 47.90 ± 3.7%.

**Figure 2:**
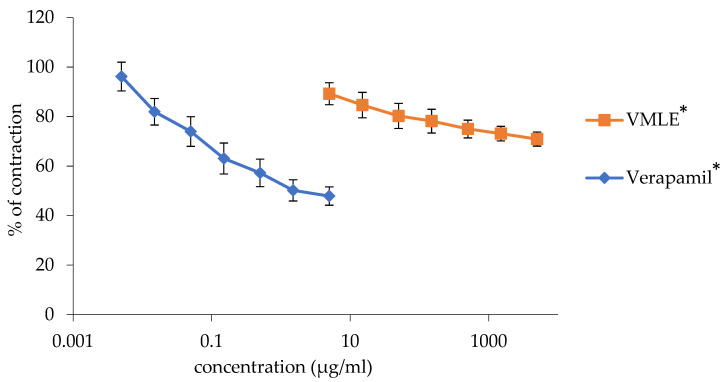
Effects of the *V. myrtillus* leaf extract (VMLE) and verapamil on KCl-induced rat ileum contractions. Contractile responses were normalized to baseline activity recorded in Tyrode’s solution (control, set as 100%) and are presented as mean ± SD from independent animals. Overall effects across the concentration range were evaluated using area under the concentration–response curve (AUC) analysis, with statistical significance assessed using a one-sample Student’s *t*-test (* *p* < 0.05).

The dose-dependent spasmolytic activities of the extract and verapamil have been represented visually in the graph below (Figure 2).

### 2.4. Effects of the V. myrtillus Extract on Acetylcholine-Induced Ileum Contractions

**Figure 3:**
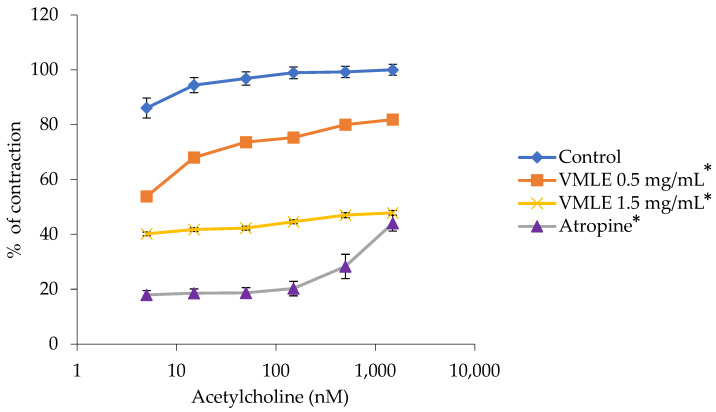
Effects of the *V. myrtillus* leaf extract (VMLE, 0.5 mg/mL and 1.5 mg/mL dose) and atropine (140 nM) on acetylcholine-induced contractions of isolated rat ileum. Contractile responses are expressed as percentages of the maximal ACh-induced contraction obtained under control conditions (set to 100%) and presented as mean ± SD. Concentration–response curves were summarized by area under the curve (AUC) analysis, and overall differences between treatments were assessed using Friedman’s test for repeated measures (* *p* < 0.05).

The *V. mytillus* extract, with doses that were added cumulatively, caused a reduction in smooth ileum muscle contractions that were previously stimulated by cumulative doses of acetylcholine (5–1500 nM). Both the *V. myrtillus* extract, in the 0.5 mg/mL and 1.5 mg/mL concentrations, as well as the positive control, atropine, caused a decrease in the acetylcholine-induced contractions. Contractions at 100%, induced by the maximum dose of acetylcholine at 1500 nM, were reduced to 81.85 ± 1.41% with 0.5 mg/mL and to 47.83 ± 0.88% with 1.5 mg/mL of this extract (Figure 3).

Atropine reduced the maximum ileum contraction from 100% to 37.55 ± 2.07%.

### 2.5. Effects of the V. myrtillus Extract on Histamine-Induced Ileum Contractions

**Figure 4:**
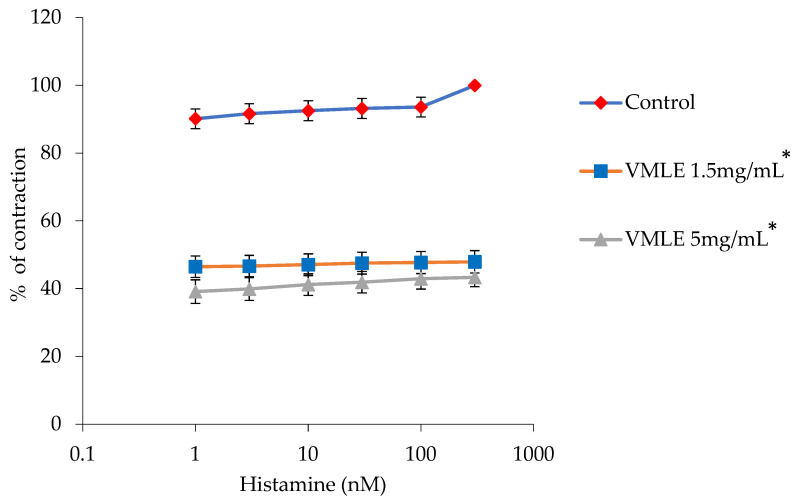
Effects of the *V. myrtillus* leaf extract (VMLE, 0.5 mg/mL and 1.5 mg/mL dose) on the histamine-induced contractions of the isolated rat ileum. Contractile responses are expressed as percentages of the maximal Histamine-induced contraction obtained under control conditions (set to 100%) and presented as mean ± SD. Concentration–response curves were summarized by area under the curve (AUC) analysis, and overall differences between treatments were assessed using Friedman’s test for repeated measures (* *p* < 0.05).

In the histamine-induced ileum contractions, histamine was given at a dose-dependent rate, with the maximum (100%) being 300 nM. The *V. myrtillus* extract, at the lower dose of 1.5 mg/mL, inhibited the histamine-caused contraction to 47.9 ± 3.3%. The higher dose of the extract (5 mg/mL) inhibited the histamine-caused contraction to 43.3 ± 2.73% (Figure 4).

### 2.6. Effects of the V. myrtillus Extract on Calcium Chloride-Induced Ileum Contractions

**Figure 5:**
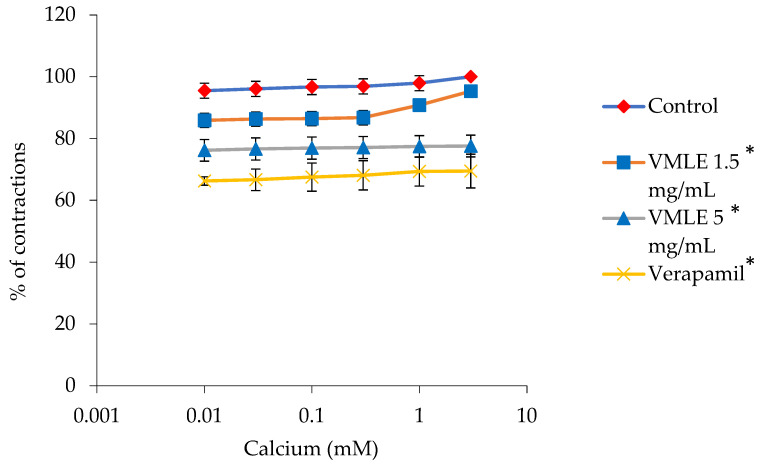
Effects of the *V. myrtillus* leaf extract (VMLE, 0.5 mg/mL and 1.5 mg/mL dose), and verapamil (0.3 μM) on the CaCl_2_-induced contractions of the isolated rat’s ileum. Contractile responses are expressed as percentages of the maximal CaCl_2_-induced contraction obtained under control conditions (set to 100%) and presented as mean ± SD. Concentration–response curves were summarized by area under the curve (AUC) analysis, and overall differences between treatments were assessed using Friedman’s test for repeated measures (* *p* < 0.05).

Figure 5 shows how calcium ions (0.01–3 mM) induced contractions of the ileum are inhibited in the presence of *V. myrtillus* extract. When the extract was used at 1.5 mg/mL, the peak contractions induced by calcium ions (taken as 100%) decreased to 95.30 ± 1.46%. Increasing the extract concentration to 5 mg/mL further lowered the contractions to 77.55 ± 3.51%. For comparison, verapamil (0.3 μM), a known calcium channel blocker, decreased the contraction response to 69.47 ± 5.45% (Figure 5).

### 2.7. Effects of the V. myrtillus Extract on the Contractions Induced by an Agonist of L-Type Ca^2+^ Channels (Bay K8644)

**Figure 6:**
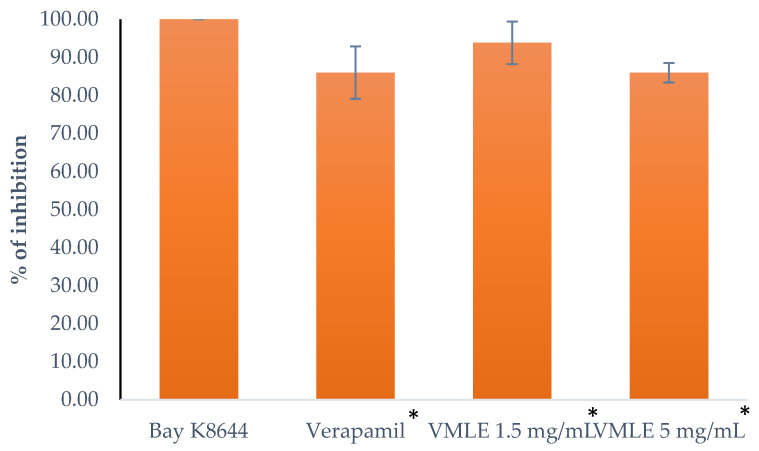
Effects of the *V. myrtillus* leaf extract (VMLE, 0.5 mg/mL and 1.5 mg/mL dose), and verapamil (10^−6^ M) on the contractions caused by Bay K8644 of the isolated rat’s ileum. Contractile responses are expressed as percentages of the maximal Bay K8644-induced contraction obtained under control conditions (set to 100%) and presented as mean ± SD. Concentration–response curves were summarized by area under the curve (AUC) analysis, and overall differences between treatments were assessed using Friedman’s test for repeated measures (* *p* < 0.05).

When measuring the effects of the *V. myrtillus* extract on the L-type Ca^2+^ channels agonist Bay K8644, the rat ileum segments were pre-treated with the extract in the 1.5 mg/mL and 5 mg/mL doses, as well as with verapamil (10^−6^ M), which was used as a control, respectively, after which the agonist was administered. The impact the tested substances made was measured by the modifications to the contractions compared to a not pre-treated ileum that had the agonist administered. The *V. myrtillus* extract at a dose of 1.5 mg/mL lowered the contractions to 93.83 ± 3.51%, the extract at the dose of 5 mg/mL lowered the contractions to 85.98 ± 2.58% of the contractions, and verapamil, used as a control, lowered the contractions to 86 ± 3.44% of the contractions (Figure 6).

### 2.8. Role of cGMP and NO on the Effect of V. myrtillus Extract on Spontaneous Contractions (ODQ and L-NAME)

**Figure 7:**
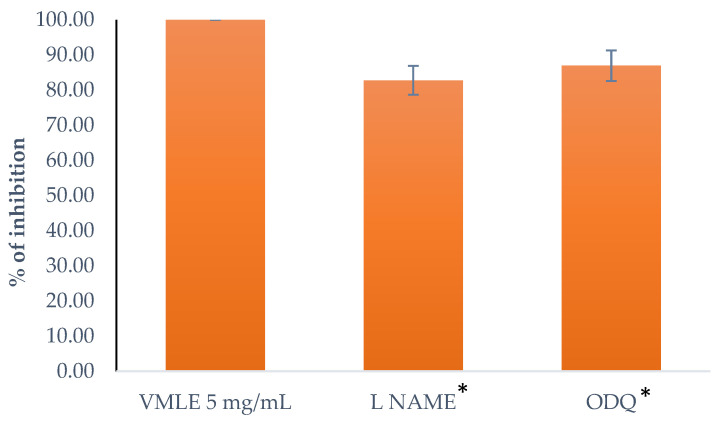
Effects of L-NAME and ODQ on the relaxant action of the *V. myrtillus* leaf extract (VMLE, 5 mg/mL, 100%) in isolated rat ileum. Data are expressed as mean ± SD of contractile activity relative to control. Statistical analysis was performed using paired Wilcoxon signed-rank tests (* *p* < 0.05).

ODQ, a soluble guanylyl cyclase suppressor in a concentration of 1 μM, and L-NAME, a nitric oxide synthase suppressor in a concentration of 10 μM, were used to investigate the role of cGMP and NO. In order to investigate the effect of the *V. myrtillus* extract, the isolated ileum segments were pre-treated for 20 min with the extract at a 5 mg/mL dose, after which ODQ and L-NAME were added, respectively. The results of the pre-treated ileum contractions after the extract were compared to those without the suppressor agents. The reduction in the potency of the extract is depicted in Figure 7. ODQ reduced the ileum contractions to 86.97 ± 4.35% and L-NAME reduced them to 82.79 ± 4.14%. The tested substances that influence the cGMP and NO pathways showed no direct effect on spontaneous ileum contractions on their own.

### 2.9. Role of K^+^ Channels on Response of V. myrtillus Extract on Spontaneous Contractions (Apamin, TRAM-34, Charybdotoxin, Glibenclamide and Quinine)

The *V. myrtillus* extract (VMLE) effect on K^+^ channels was measured by applying the extract in a 5 mg/mL dose to an isolated rat ileum that was pre-treated for 20 min with the tested K^+^ inhibitor (Apamin, TRAM-34, Charybdotoxin, Glibenclamide or Quinine), after which the effects of the extract were recorded.

**Figure 8:**
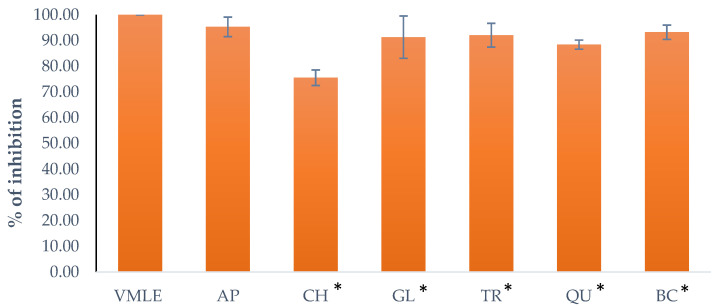
Effects of L-NAME and ODQ on the relaxant action of the *V. myrtillus* leaf extract (VMLE, 5 mg/mL, 100%) on the contractions modified by Apamin (AP), Charybdotoxin (CH), Glibenclamide (GL), Tram-34 (TR), Quinine (QU) and BaCl2 (BC). Data are expressed as mean ± SD of contractile activity relative to control. Statistical analysis was performed using paired Wilcoxon signed-rank tests (* *p* < 0.05).

Apamin (1 μM, AP), a selective voltage-activated K^+^ channel and small-conductance Ca^2+^ inhibitor, produced a reduction in the contractions to 95.34 ± 3.81%. BaCl_2_ (0.9 mM, BC), an inhibitor of the inward rectifier K^+^ channel, produced a reduction in the contractions to 93.26 ± 2.79%. Charybdotoxin (0.01 μM, CH), a specific inhibitor of intermediate and large-conductance Ca^2+^ activated K^+^ channels, produced a reduction in the contractions to 75.57 ± 3.02%. Glibenclamide (10 M, GL), an inhibitor of ATP-sensitive K^+^ channels, produced a reduction in the contractions to 91.34 ± 8.22%. TRAM-34 (1 μM, TR), a selective inhibitor of intermediate conductance Ca^2+^ activated K^+^ channels, produced a reduction in the contractions to 92.11 ± 4.6%. Quinine (10 μM, QU), an inhibitor of voltage-sensitive K^+^ channels, produced a reduction in the contractions to 88.45 ± 1.76% %. Figure 8 represents the individual effects of the given substances compared to the effect of the extract alone on the same tissue sample.

## 3. Discussion

Although bilberry leaves are increasingly recognized as an alternative source of bioactive natural products with medicinal and nutritional relevance [7], studies addressing the quantification of phenolic compounds in the leaves of *V. myrtillus* remain limited. These leaves are considered a rich source of phenolic compounds, particularly phenolic acids and flavonoids [3,7,15,16]. In accordance with our HPLC-DAD findings, chlorogenic acid and isoquercitrin were identified as the predominant phenolic acid and flavonoid in the *V. myrtillus* leaf extract, in agreement with previous reports [15,17]. In the study by Liu et al. (2014) [16] on *V. myrtillus* leaves from Finland, chlorogenic acid was also reported as the predominant phenolic acid, whereas quercetin-3-*O*-glucuronide was the major flavonoid. Quercetin-3-*O*-glucuronide was also the predominant flavonoid in *V. myrtillus* leaves from Italy [18]. Variations in phenolic content reported in different studies on *V. myrtillus* leaves can be attributed to the geographical origin of the plant material, abiotic environmental factors, harvesting time, and differences in extraction methodologies [3,15,18].

As a pleasant-tasting fruit, the berries of *V. myrtillus* have more frequently been employed in traditional medicine for gastrointestinal symptomatology [19,20], while the leaves have also been occasionally mentioned in this context [10]. However, neither the fruit nor the leaves have so far been experimentally validated for spasmolytic activity in gastrointestinal disorders. Our results provide experimental support for this traditional use, as the leaf extract of *V. myrtillus* demonstrated a significant concentration-dependent spasmolytic effect on isolated rat ileum. To the best of our knowledge, this is the first confirmation of spasmolytic activity of a preparation derived from any *Vaccinium* species.

Regarding the mechanism of spasmolytic activity, noteworthy are the effects of the extract on spontaneous contractions of the isolated ileum, as well as its noteworthy influence on Ca^2+^ channels, cGMP, histamine- and NO-mediated contractions. The impact on K^+^ channel-mediated contractions was pronounced, with the most prominent effect observed on intermediate- and large-conductance Ca^2+^-activated K^+^ channels, whereas the effect of small-conductance Ca^2+^- and voltage-activated K^+^ channels was deemed not statistically significant. When interpreting these results, it should be noted that they mainly reflect pharmacodynamic effects and do not provide direct evidence for the involvement of specific signaling pathways. Collectively, these results support the hypothesis that the complex chemical composition of plant products may produce multiple pharmacodynamic effects, which is particularly valuable given the challenge posed by the presence of multiple molecular targets in smooth muscle contraction disorders associated with spasms [21].

The major phenolic compounds identified in the *V. myrtillus* extract are possible mediators of its spasmolytic activity. Literature reports indicate that several of these compounds possess spasmolytic properties on ileum contractions [22,23,24,25,26,27]. Among them, chlorogenic acid, which was the most abundant phenolic compound in our HPLC quantification, is of particular interest. This compound has been frequently associated with strong effects on ileum contractions, and several plant species with notable spasmolytic activity also contained chlorogenic acid as their predominant phenolic constituent [25,26]. Similarly, isoquercitrin, identified as the dominant flavonoid, along with quercitrin, rutin, and quercetin, detected in lower amounts, are known spasmolytic agents. Namely, previous studies demonstrated their activity on isolated guinea pig ileum strips, although no reports are available on the effects of isoquercitrin, quercitrin, or quercetin on isolated rat ileum [22,23,24]. Conversely, rutin has also been reported as a spasmogenic factor in rat ileum contractions [27]. The main spasmolytic constituents could be identified through directed fractionation of the extract, although crude extracts often show greater activity than isolated fractions, likely due to synergistic interactions among bioactive compounds [28]. Among phytochemicals reported to exhibit spasmolytic activity, flavonoids are the second most frequently occurring class of secondary metabolites, after monoterpenoids [14].

Concerning the spasmolytic effects of tannin-rich plants, the available data remains limited. In experiments on isolated ileum from mice, tannic acid exhibited a very mild spasmolytic effect [29]. In contrast, it has been demonstrated that a purified extract of *Castanea sativa* wood (i.e., a water-soluble fraction containing 77.8% pure tannins) was able to induce relaxation of guinea pig ileum and proximal colon smooth muscle spasms triggered by different mechanisms [30]. Investigation of the molecular mechanism revealed a certain selectivity of this extract, as it inhibited cholinergic activity without directly interfering with serotonergic receptor activation. The authors of that study highlighted the therapeutic potential of this tannin-rich extract in the treatment of diarrhea, attributing its efficacy to a synergistic interaction between its antispasmodic and antimicrobial effects. Furthermore, the potential of tannins in gastrointestinal disorders is supported by their inability to cross the intestinal barrier, as they mainly exert localized pharmacological effects within the digestive tract, thereby minimizing systemic actions [31].

## 4. Materials and Methods

### 4.1. Plant Materials

Bilberry leaves (*V. myrtillus*) were obtained from the manufacturing sector of the Institute for Medicinal Plant Research “Dr. Josif Pančić”, Belgrade, Serbia. The leaves were stored in a dark, dry place prior to further analysis. The plant material has been authenticated by the quality control sector of the Institute for Medicinal Plant Research “Dr. Josif Pančić”, Belgrade (product serial number: 52981224).

### 4.2. Extraction Procedure

The double percolation technique, using a 50% ethanol-water solution as the solvent, was employed to prepare the extract. Percolation was carried out at room temperature with a drug-to-solvent ratio of 1:5 g/mL. The resulting extract was evaporated using a vacuum evaporator (Buchi rotavapor R-114) until ethanol removal. The prepared extract was stored in dark bottles in the refrigerator at 4 °C until the next stage of processing.

### 4.3. Spray Dried Powder Preparation

The liquid extract of bilberry leaves was spray-dried without the addition of carriers according to our previously published procedure [17]. The extract was heated to 40 °C and mixed with a magnetic stirrer to ensure constant homogenization, then microencapsulated in a LabtexESDTi spray dryer (Labtex, Huddersfield, UK) under the following conditions: inlet temperature of 130 ± 5 °C, outlet temperature of 70 ± 5 °C, liquid feed rate of 11 mL/min, spraying air flow rate of 75 m^3^/h, and atomization pressure of 2.5 bar. Prior to analysis, the powders were stored in glass bottles in desiccators at room temperature.

### 4.4. HPLC Analysis

The chromatographic analyses were performed using an Agilent 1260 RR HPLC system (Agilent Technologies, Waldbronn, Germany) equipped with a diode array detector (DAD), following the procedures described by [17]. Separation was carried out on a Zorbax SB-C18 reversed-phase analytical column (Agilent, 150 × 4.6 mm i.d., 5 μm particle size). The mobile phase consisted of solvent A (1% *v*/*v* orthophosphoric acid in water) and solvent B (acetonitrile), applied under the following gradient program: 0–2.6 min, 90–85% A; 2.6–8 min, 85% A; 8–10.8 min, 85–80% A; 10.8–18 min, 80% A; 18–23 min, 80–70% A; 23–25 min, 70–50% A; 25–27 min, 50–30% A; 27–29 min, 30–10% A; 29–31 min, 10–0% A; and 31–34 min, 0% A. The flow rate was maintained at 0.8 mL/min, and the column temperature was set to 40 °C. The injection volume was 8 μL. Detection was carried out at 260, 280, 320, and 360 nm. Compound identification was based on the comparison of retention times and UV spectra with those of authentic standards, while quantification was performed using external calibration curves. The contents of phytocompounds were expressed as milligrams per gram of powdered extract dry weight (mg/g DW).

### 4.5. Determination of Total Polyphenol Content

The total polyphenol content was determined using the Folin–Ciocalteu spectrophotometric assay. Absorbance values of the sample and standard solutions were recorded at 725 nm. Quantification was performed using a calibration curve constructed with gallic acid as the reference standard. All measurements were carried out in triplicate. The results were expressed as milligrams of gallic acid equivalents per gram of dry extract (mg GAE/g DW) [32].

### 4.6. Determination of Total Tannin Content

The total tannin content was determined using a modified Folin–Ciocalteu spectrophotometric method, based on a previously described procedure, following selective removal of tannins by adsorption onto an insoluble matrix, polyvinylpolypyrrolidone (PVPP). The tannin content was calculated as the difference between total polyphenols and non-tannin polyphenols. Quantification was performed using a calibration curve constructed with gallic acid as the reference standard. All measurements were carried out in triplicate. The results were expressed as mg GAE/g DW [32].

### 4.7. Experimental Animal Housing

All experimental procedures were conducted in compliance with the European Directive 2010/63/EU governing animal experimentation. Ethical approval was granted by the Ethical Committee of the Faculty of Medicine, University of Niš (decision no. 01-206-7). Male Wistar albino rats, 10–12 weeks old and weighing 200–250 g, were obtained from the Vivarium of the Research Centre for Biomedicine, Faculty of Medicine, University of Niš. Prior to the start of the experiments, animals were housed individually for one week to allow for acclimatization. During this period, they were kept in stainless steel cages under standard laboratory conditions, with ambient temperatures maintained between 20 and 24 °C and a 12 h light–dark cycle. Food and water were available ad libitum throughout the study, except for the 24 h immediately before experimentation, when food access was withheld.

### 4.8. Methodology for Isolated Rat Ileum Contractions

#### 4.8.1. The Isolation and Placement of Rat Ileum

At the beginning of the experiment, the tested rat is placed in a closed chamber and exposed to ether vapors. With the rat successfully in a state of anesthesia, its chest is opened and the aorta is cut. After this, the ileum is extracted and cut into 2 cm strips, with the attached mesentery being removed in the process. Next, the ileum segments are placed into an organ bath that contains 20 mL of physiological solution for isolated intestines (Tyrode’s solution). The temperature of the bath is at 37 °C and the chamber is receiving a constant influx of a blend of oxygen and carbon dioxide. (95% and 5%). The composition of the Tyrode’s solution included the following constituents: 150 mM NaCl, 2.7 mM KCl, 2 mM MgCl_2_, 12 mM NaHCO_3_, 0.4 mM NaH_2_PO_4_, 1.8 mM CaCl_2_, and 5.5 mM glucose. Half an hour before the start of the experiment, the ileum was stabilized in the bath [33].

Alterations in small intestine contractility were monitored using a transducer (Transducer–TSZ-04-E, Experimetria Ltd., Budapest, Hungary), and the acquired data were processed utilizing the SPEL Advanced ISOSYS Data Acquisition System software.

#### 4.8.2. The Effects of the *V. myrtillus* Extract on Acetylcholine-Induced Contractions of Rat Ileum

In order to represent the effect that the *V. myrtillus* extract had on the acetylcholine-induced contractions, a concentration-response curve was first established by gradually adding increasing concentrations of acetylcholine (5, 15, 50, 150, 500, and 1500 nM) to the ileum tissue following an initial adaptation period. Afterward, the tissue was rinsed using Tyrode’s solution to allow spontaneous contractions to return to baseline levels.

Once the ileum was washed, a test solution containing the extract at 1.5 mg/mL was added to the organ bath. After a 5-min incubation, the acetylcholine concentration series was repeated to generate a new response curve representing acetylcholine’s contractile activity in the presence of the extract.

Following this, the tissue was again washed with Tyrode’s solution to restore spontaneous activity and was then exposed to a higher extract concentration (5 mg/mL), using the same procedure to assess its effect on acetylcholine-induced contractions.

The spasmolytic potential of the extract was evaluated by comparing the percentage of contraction induced by acetylcholine alone to that in the presence of the extract. The data acquired this way was later presented as dose–response curves [33]. Atropine (140 nM), a non-selective blocker of muscarinic receptors, served as the positive control.

#### 4.8.3. The Effects of the *V. myrtillus* Extract on Histamine-Induced Contractions of Rat Ileum

In this set of experiments, ileum preparations were first allowed to adapt for 30 min before contractions were induced. Gradually increasing concentrations of histamine (1, 3, 10, 30, 100, and 300 nM) were then added to the organ bath to trigger ileum contractions. A control concentration-response curve was generated, showing the relationship between histamine concentration and the percentage of contraction.

After the control data was collected, the tissues were then rinsed with Tyrode’s solution to reestablish stable spontaneous contractions. The extract at 1.5 mg/mL was then added to the bath, and after five minutes, the same concentrations of histamine were applied again (1–300 nM). This procedure was then repeated using the extract at a concentration of 5 mg/mL.

To evaluate the extract’s spasmolytic activity, contraction curves were produced showing the effect of histamine (%) both in the presence of the extract at 1.5 mg/mL and 5 mg/mL, as well as without the extract (control) [33].

#### 4.8.4. The Role of Calcium Channels in the Effect of the *V. myrtillus* Extract

To further clarify the mechanism behind the effects of the extract, the extract’s activity was compared to that of established receptor activators. The influence of the extract on calcium chloride-induced contractions in rat ileum was assessed. For this experiment, ileum segments were adapted in a calcium-free solution until stable spontaneous contractions were observed. When they were observed, increasing concentrations of CaCl_2_ (0.01, 0.03, 0.1, 0.3, 1, and 3 mM) were added to the bath, and the contractile responses were recorded. A concentration-response curve was generated from this data. The tissues were then washed repeatedly until stable spontaneous contractions returned.

After the experiments with only CaCl_2_, the extract at 1.5 mg/mL was introduced, and after five minutes, the same concentrations of CaCl_2_ were applied. The process was repeated using a 5 mg/mL concentration of the extract. Curves illustrating CaCl_2_-induced contractile responses in the presence of the extract at both concentrations were created. Verapamil (0.3 µmol/L) was used as a reference standard.

The spasmolytic properties of the extract were represented through concentration-response curves that showed the contractile activity of CaCl_2_ (%) in the presence of the extract, and these were compared to the control data where only CaCl_2_ was applied (without the extract) [33].

The extract’s effect on potassium chloride-induced contractions in rat ileum was also evaluated. After an adaptation phase, KCl (80 mM) was used to induce sustained contractions. These tonic contractions were progressively inhibited by cumulative doses of the extract (0.005–5 mg/mL), added at 15-min intervals. The degree of inhibition was determined by calculating the area under the curve differences from baseline, expressed as a percentage of KCl-induced contraction reduction. Verapamil, as a positive control, was tested at concentrations ranging from 0.015 to 1.5 μg/mL [33].

Finally, the action of the extract on contractions induced by Bay K8644, an L-type Ca^2+^ channel agonist, was studied. Ileum segments were pre-incubated for 15 min with the extract (1.5 or 5 mg/mL) or verapamil (10^−6^ M) before Bay K8644 was added. The contractile responses obtained in the presence of the extract or verapamil were compared to the response produced by Bay K8644 alone (set as 100% control). Verapamil was used here as a positive control [34].

#### 4.8.5. The Role of NO and cGMP in the Effect of the *V. myrtillus* Extract

In this section of the study, L-NAME (N*ω*-Nitro-L-arginine methyl ester), a nitric oxide synthase (NOS) inhibitor, and ODQ (1H-[1,2,4]oxadiazolo [4,3-a]quinoxalin-1-one), a selective and potent soluble guanylyl cyclase inhibitor, were used. Ileum segments were pretreated separately with 10 µM L-NAME or 1 µM ODQ 20 min prior to adding the extract, in order to investigate the role of the nitric oxide (NO)/cGMP signaling pathway in the extract (5 mg/mL) induced relaxation of spontaneous contractions. The results were then compared to those obtained when the extract was applied alone on the same organ tissue [34].

#### 4.8.6. The Role of K^+^ Channels in the Effect of the *V. myrtillus* Extract

In this experimental setup, several compounds were utilized individually: apamin (1 µM), a selective blocker of small-conductance Ca^2+^- and voltage-activated K^+^ channels (SKCa); BaCl_2_ (0.9 mM), an inhibitor of inward rectifier K^+^ channels (KIR); charybdotoxin (0.01 µM), a specific blocker of both intermediate- and large-conductance Ca^2+^-activated K^+^ channels; glibenclamide (10 µM), an ATP-sensitive K^+^ channel (KATP) inhibitor; TRAM-34 (1 µM) [1-[(2-chlorophenyl) diphenylmethyl]-1H-pyrazole], which selectively inhibits intermediate-conductance Ca^2+^-activated K^+^ channels (IKCa); and quinine (10 µM), a voltage-sensitive K^+^ channel blocker.

To assess their effects on ileum contractions, each of these agents was introduced individually at the stated concentration 20 min prior to the addition of the extract (5 mg/mL). The aim was to observe how these substances influenced the extract’s contractile effect. The outcomes were compared to those obtained when the extract was applied alone to the same organ tissue. Each of these compounds followed the same testing methodology and was compared only to results obtained by the presence or absence of the given substance [34].

### 4.9. Statistical Analysis

Raw contractile force recordings were normalized within each preparation to its own reference response in order to minimize inter-animal variability. For concentration–response experiments, responses were normalized to the maximal agonist-induced contraction obtained under control conditions, which was defined as 100%. Concentration–response relationships were summarized by calculating the area under the concentration–response curve (AUC) for each animal using the trapezoidal rule applied to responses plotted against log-transformed agonist concentrations. This approach provides a single quantitative measure of the overall response across the tested concentration range while avoiding multiple comparisons at individual concentration points.

For experiments assessing the inhibitory effects of the extract on spontaneous activity or on sustained potassium chloride (KCl)-induced contractions, responses were expressed as percentages of the corresponding control contraction, which was defined as 100%. In these experiments, AUC values or mean percentage inhibitions were compared against the theoretical no-effect value (100%) using a one-sample Student’s *t*-test or its non-parametric equivalent, as appropriate.

For mechanistic experiments investigating the involvement of specific signaling pathways or ion channels (including L-NAME, ODQ, and potassium channel blockers), the effects of the extract were compared in the absence and presence of the respective inhibitor within the same preparations. These comparisons were performed using paired statistical analyses, as each preparation served as its own control. Accordingly, paired non-parametric comparisons were conducted using the Wilcoxon signed-rank test.

All statistical analyses were performed using the animal as the independent experimental unit. For comparisons involving three or more experimental conditions within the same animals, overall differences were evaluated using Friedman’s test for repeated measures. Where appropriate, post hoc pairwise comparisons versus control were conducted using Wilcoxon signed-rank tests with Holm correction applied to account for multiple testing.

Data are presented as mean ± SD unless otherwise stated. Statistical significance was accepted at *p* < 0.05. All statistical analyses were performed using SPSS version 20.0 software (SPSS Inc., Chicago, IL, USA).

## 5. Conclusions

This study provides a valorization of *Vaccinium myrtillus* L. (Ericaceae) leaves as an underutilized medicinal material and a rich source of polyphenol compounds. Phytochemical profiling by HPLC-DAD revealed that they contain phenolic acids and flavonoids, with chlorogenic acid and isoquercitrin being the dominant representatives, respectively.

Guided by the traditional use of the leaves as an antispasmodic remedy, ethnopharmacological validation was conducted on isolated rat ileum, confirming their spasmolytic activity. After verifying the activity on spontaneous ileum contractions, deeper insight into the mechanism of action was obtained through thirteen complementary experimental models, including potassium chloride-, acetylcholine-, histamine-, calcium chloride-, and Bay K8644-induced contractions, as well as contractions in the presence of ODQ and L-NAME to assess the roles of the cGMP and NO pathways, and contractions in the presence of apamin, BaCl_2_, TRAM-34, charybdotoxin, glibenclamide, and quinine to assess the role of potassium channels.

The spasmolytic activity was confirmed to involve significant influence on Ca^2+^ channel-, histamine-, cGMP-, and NO-mediated contractions. The effect on K^+^ channel-mediated contractions was pronounced, with the most notable performance observed in relation to intermediate- and large-conductance Ca^2+^-activated K^+^ channels.

Altogether, the results of this study indicate the potential of *V. myrtillus* leaves in the phytotherapy of gastrointestinal disorders. To substantiate this potential and ensure their efficacy and safety, further in vivo and clinical studies are required.

## Figures and Tables

**Table 1:** Phytochemical composition of the investigated *V. myrtillus* leaf extract.

Chemical Structure	Compound Name	Content (mg/g Dry Extract)
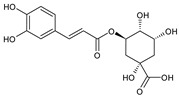	Chlorogenic acid	52.06 ± 1.08
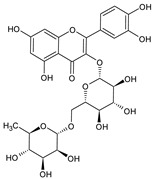	Rutin	7.02 ± 0.16
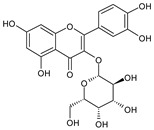	Hyperoside	11.45 ± 0.37
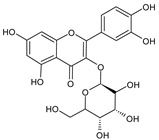	Isoquercitrin	35.28 ± 0.83
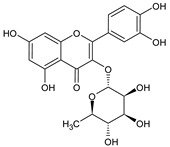	Quercitrin	9.27 ± 0.29
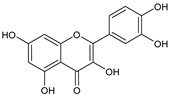	Quercetin	1.16 ± 0.03
Total polyphenol content		122.79 ± 10.35 *
Total tannin content		71.87 ± 5.43 *

* Values are expressed as milligrams of gallic acid equivalents per gram of dry extract.

## Data Availability

Data are contained within the article.
